# Determination of Fluid Density and Viscosity by Analyzing Flexural Wave Propagations on the Vibrating Micro-Cantilever

**DOI:** 10.3390/s17112466

**Published:** 2017-10-27

**Authors:** Deokman Kim, Seongkyeol Hong, Jaesung Jang, Junhong Park

**Affiliations:** 1 Department of Mechanical Engineering, Hanyang University, Seoul 04763, Korea; deokman@hanyang.ac.kr; 2 Department of Mechanical Engineering, Ulsan National Institute of Science and Technology (UNIST), Ulsan 44919, Korea; miffy0691@naver.com

**Keywords:** density, viscosity, wave propagation analysis, cantilever sensor, fluid-structure interaction

## Abstract

The determination of fluid density and viscosity using most cantilever-based sensors is based on changes in resonant frequency and peak width. Here, we present a wave propagation analysis using piezoelectrically excited micro-cantilevers under distributed fluid loading. The standing wave shapes of microscale-thickness cantilevers partially immersed in liquids (water, 25% glycerol, and acetone), and nanoscale-thickness microfabricated cantilevers fully immersed in gases (air at three different pressures, carbon dioxide, and nitrogen) were investigated to identify the effects of fluid-structure interactions to thus determine the fluid properties. This measurement method was validated by comparing with the known fluid properties, which agreed well with the measurements. The relative differences for the liquids were less than 4.8% for the densities and 3.1% for the viscosities, and those for the gases were less than 6.7% for the densities and 7.3% for the viscosities, showing better agreements in liquids than in gases.

## 1. Introduction

Viscosity and density are the fundamental properties of a fluid, and their measurements are important in many applications, from the quality control of combustible gases [1] to blood coagulation tests [2]. Various kinds of cantilever sensors have been developed to measure the viscosity and density of fluids [1,2,3,4,5,6,7,8,9,10], which are usually based on the resonance of the cantilever [1,3,4,5,7,8,9,10]. That is, the density and viscosity of a fluid are obtained from a change in resonant frequency and peak width (or quality factor) when the cantilever is immersed in the fluid. These cantilevers were approximated as vibrating spheres in a viscous environment [5,9], or the hydrodynamic function [11] was used to account for the real geometry of the cantilevers [4,10], which allows the multimode analysis, but places a restriction in the frequency range and an added mass on the cantilever.

In our previous study [12], we presented simultaneous measurements of the mass and position of micro-beads attached to the vibrating cantilever in fluids. Wave propagations along the cantilever depended on the interaction between the beads, the cantilever, and the surrounding fluid. The change in the wave propagations was used for the measurements. In the present study, we propose an alternative method to determine the density and viscosity of a fluid using a wave propagation analysis for piezoelectrically excited microscale- and nanoscale-thickness cantilevers depending on their sensitivities in a gas or a liquid. The viscosity and density of the fluid can then be obtained by measuring the change in the wavenumber induced by the fluid-structure interaction. The fluid-structure interactions and the wave propagation on the cantilevers partially and totally immersed in viscous fluids were analyzed to calculate the transfer functions of the microscale- and nanoscale-thickness cantilevers in liquids and gases, respectively. Both viscosity and density were then determined and compared with the known values.

The measured transfer function and wavenumber of the cantilever presented more reliable information over a wide range of frequencies, whereas commonly used resonance-based methods require a frequency range close to the resonant frequencies and are subject to a significant amount of uncertainties in case of liquids due to their low quality factors [12]. In this regard, the use of wave propagation characteristics is advantageous because it does not require the accurate determination of natural frequencies, thereby providing a reliable method to find the properties of liquids.

## 2. Theoretical Analysis

### 2.1. Fluid-Structure Interaction

**Figure 1:**
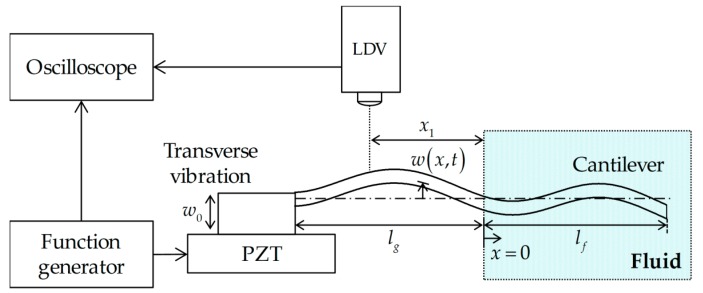
A schematic of the experimental setup, which consists of a micro-cantilever partially immersed in a fluid, a single-point laser Doppler vibrometer (LDV), a function generator, and a lead zirconate titanate (PZT). The vibration velocity of the cantilever was measured at $x=-x_{1}$ via the LDV. The figure shows standing wave patterns of the partially immersed cantilever in the fluid at the fourth resonance frequency.

When a cantilever is excited in a fluid, the fluid-structure interaction leads to the resistance and dissipation of the wave propagation on the cantilever (Figure 1). The interaction force can be given as [11,13]:

(1) $$F\left(x,\omega \right)=\frac{\pi }{4}\rho \omega^{2}b^{2}\Gamma \left(\omega \right)w\left(x,\omega \right)$$

where *ρ* is the density of the fluid, *ω* is the radial frequency, *b* is the width, and Γ(ω) is the hydrodynamic function of the cantilever. The hydrodynamic function can be expressed as $\Gamma \left(\omega \right)=\Gamma_{r}\left(\omega \right)+i\Gamma_{i}\left(\omega \right)$, where i is the imaginary number, assuming that the deflection of the cantilever can be shown in the form of $w\left(x,t\right)=\mathrm{Real}\left\{\hat{w}\left(x\right)e^{-i\omega t}\right\}$. The hydrodynamic function depends on the Reynolds number [14], Re=ρωb2/4η, where η is the viscosity of the fluid. The hydrodynamic function of a cantilever with a rectangular cross-section ($\Gamma_{\mathrm{rect}}$) needs to be revised through numerical procedures from that with a circular cross-section ($\Gamma_{\mathrm{circ}}$). The hydrodynamic function of the rectangular beam can then be expressed as [11]:

(2) Γrect(ω)=Ω(ω)Γcirc(ω)=Ω(ω)[1+4iK1(−iiRe)iReK0(−iiRe)]

in the range Re∈[10−6,104], where $K_{0}$ and $K_{1}$ are the modified Bessel functions of the third kind, and the correction function, respectively, and Ω is expressed as fractional polynomial functions of log10Re.

### 2.2. Flexural-Wave Propagation on the Cantilever Immersed in a Fluid

For the cantilever immersed in a fluid, the quality factor depends on the cantilever bending stiffness and the Reynolds number, and both microscale- and nanoscale-thickness cantilevers were used in this study. When a microscale-thickness cantilever was completely immersed in a liquid, the decay rate of the wave propagation on the cantilever was too large to induce standing waves. Therefore, the microscale-thickness cantilevers were partially immersed in liquids to improve the sensitivity of dynamic property measurements, whereas the nanoscale-thickness cantilevers were fully immersed in gases.

If the cantilever has a small thickness and width compared to the lengthwise dimension, the effects of the shear deformation and the rotary inertia are negligibly small compared to those of the bending deformation. The equation of motion for a section of a cantilever immersed in a fluid can be expressed as [15]:

(3) $$D\frac{\partial^{4}w\left(x,t\right)}{\partial x^{4}}+M_{b}\frac{\partial^{2}w\left(x,t\right)}{\partial t^{2}}=F\left(x,t\right)$$

where *D* is the bending stiffness, *w* is the deflection, *x* is the lengthwise coordinate with the origin at the interface between vacuum (or air) and fluid, and *M_b_* is the mass per unit length of the cantilever. Assuming harmonic vibration, the response of the cantilever partially immersed in a fluid can be given as:

(4) $$\begin{array}{l} \hat{w}(x)=\left({\hat{A}}_{1}e^{-i{\hat{k}}_{b}x}+{\hat{A}}_{2}e^{i{\hat{k}}_{b}x}+{\hat{A}}_{3}e^{{\hat{k}}_{b}x}+{\hat{A}}_{4}e^{-{\hat{k}}_{b}\left(x+l_{g}\right)}\right)\left[1-H\left(x\right)\right]\\ +\left({\hat{A}}_{5}e^{-i{\hat{k}}_{bf}x}+{\hat{A}}_{6}e^{i{\hat{k}}_{bf}x}+{\hat{A}}_{7}e^{{\hat{k}}_{bf}(x-l_{f})}+{\hat{A}}_{8}e^{-{\hat{k}}_{bf}x}\right)H\left(x\right) \end{array}$$

where ${\hat{A}}_{n}\left(n=1,2,...,8\right)$ are the coefficients to be determined by the boundary conditions, $l_{g}$ and $l_{f}$ are the partial cantilever lengths in vacuum and fluid, respectively, *H* is the Heaviside step function, and ${\hat{k}}_{b}$ and ${\hat{k}}_{bf}$ are the wavenumbers in vacuum and fluid, respectively.

After substituting Equations (1) and (4) into (3), the wavenumbers of the cantilever beam in a fluid is derived from Equation (3) as:

(5) $${\hat{k}}_{bf}={\left\{\omega^{2}\left(4M_{b}+\pi \rho b^{2}\Gamma_{r}-i\pi \rho b^{2}\Gamma_{i}\right)/4\hat{D}\right\}}^{1/4}$$

To solve for the unknown coefficients, ${\hat{A}}_{\;1}\sim {\hat{A}}_{\;8}$, the boundary conditions were applied as:

(6) $$\begin{array}{l} \hat{w}(-l_{g})=w_{0},\;\frac{\partial \hat{w}(-l_{g})}{\partial x}=\frac{\partial^{2}\hat{w}(l_{f})}{\partial x^{2}}=\frac{\partial^{3}\hat{w}(l_{f})}{\partial x^{3}}=0,\\ \hat{w}(0^{-})=\hat{w}(0^{+}),\;\frac{\partial \hat{w}(0^{-})}{\partial x}=\frac{\partial \hat{w}(0^{+})}{\partial x},\;\frac{\partial^{2}\hat{w}(0^{-})}{\partial x^{2}}=\frac{\partial^{2}\hat{w}(0^{+})}{\partial x^{2}},\;\frac{\partial^{3}\hat{w}(0^{-})}{\partial x^{3}}=\frac{\partial^{3}\hat{w}(0^{+})}{\partial x^{3}}. \end{array}$$

Then the transfer function between the vibration response at $x=-x_{1}$ and the input excitation at $x=-l_{g}$ can be calculated as [16]:

(7) $$\Lambda e^{i\phi }={\hat{w}}_{1}(-x_{1})/w_{0}$$

where *Λ* and φ are the amplitude and the phase of the transfer function, respectively. For the cantilevers partially or fully immersed in a viscous fluid, the density and viscosity were obtained by the wavenumber, Equation (5).

The estimation of the fluid properties from the fluid structure interaction above requires a numerical process. The numerical method was applied to estimate the effects of fluid loading on the wave propagations on the cantilever. The transfer function in Equation (7) is a function of the complex wavenumber, ${\hat{k}}_{bf}=k_{bfr}-ik_{bfi}$, influenced by the fluid density and viscosity. The fluid properties were estimated by a two-step numerical process. The complex wavenumber was calculated using the transfer function. The measured transfer function, $\Lambda e^{i\phi }$, was compared with the predictions. The Newton-Raphson method was used to numerically solve the quadratic matrix equation as:

(8) [kbfrkbfi]j+1=[kbfrkbfi]j−[Re{∂w^1(−x1)∂kbfr,∂w^1(−x1)∂kbfi}Im{∂w^1(−x1)∂kbfr,∂w^1(−x1)∂kbfi}]−1[Re{w^1(−x1)−w0Λeiϕ}Im{w^1(−x1)−w0Λeiϕ}]

where j is the iteration number. To estimate the fluid properties, the obtained complex wavenumber, ${{\hat{k}}^{\prime }}_{bf}$, of the cantilever interacting with the fluid was compared with the prediction using a similar matrix:

(9) [ρη]j+1=[ρη]j−[Re{∂k^bf∂ρ,∂k^bf∂η}Im{∂k^bf∂ρ,∂k^bf∂η}]−1[Re{k^bf−k^′bf}Im{k^bf−k^′bf}]

Consequently, the fluid density and viscosity were simultaneously estimated from the measured complex wavenumber.

## 3. Experimental Setup

Figure 1 shows the schematic of the experimental setup where the free end of the cantilever is partially immersed in a fluid. The fabrication of the nanoscale-thickness cantilevers was described in previous work [12], and the microscale-thickness cantilever was purchased from a company (ALOAL-TL, Probes, Seoul, Korea). The cantilever chip was clamped on a lead zirconate titanate (PZT; Physik Instrumente, Karlsruhe, Germany) sheet providing excitation. A noise with the bandwidth of 1 MHz and peak-to-peak voltage of 3 V was generated by a function generator (33500B, Agilent) and applied to the PZT sheet. The vibration velocity of the cantilever was measured at the clamped boundary and the one-fifth position of the beam through a single-point laser Doppler vibrometer (Polytec, Dexter, MI, USA) [12]. The voltages from the function generator and output voltage from the laser Doppler vibrometer (LDV) measurement were used to obtain the transfer function.

**Figure 2:**
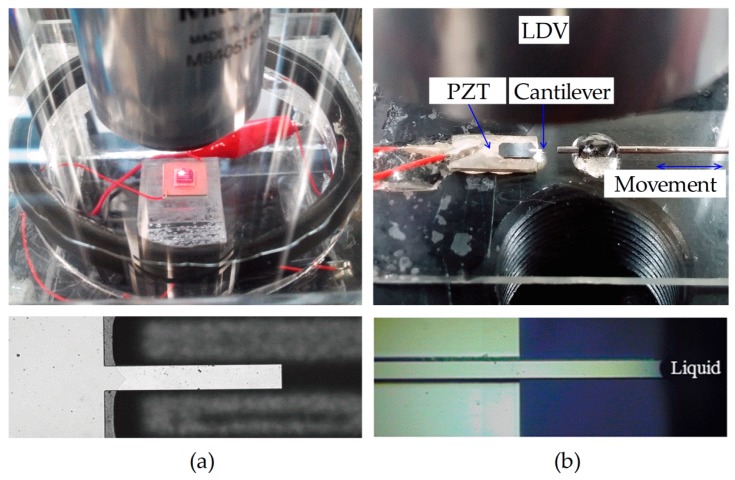
(**a**) Experimental setup for the measurement of the gas properties using a microfabricated nanoscale-thickness cantilever in a gas chamber and (**b**) the liquid properties using a microscale-thickness cantilever partially immersed in liquid.

Figure 2a,b show the experimental systems and the magnified views of the nanoscale-thickness cantilever [12] (150 × 10 × 0.20 μm^3^, 0.70 ± 0.20 ng) fully immersed in a gas and the microscale-thickness cantilever (458 × 51 × 2.0 μm^3^, 109 ± 58 ng) partially immersed in a liquid, respectively. Considering the size of the cantilever chips, the nanoscale-thickness cantilever chip (5 × 5 × 0.7 mm^3^) was attached to a 10 × 10 × 0.5 mm^3^ PZT chip, while the microscale-thickness cantilever chip (1.5 × 3.5 × 0.3 mm ^3^) was fixed to a 4 × 6 × 0.5 mm^3^ PZT chip. Two wires were connected to the PZT chips to apply the excitation voltage. The nanoscale-thickness cantilever chip with the PZT chip was placed inside a gas chamber (70 × 65 × 30 mm^3^; Figure 2a) which was composed of two throttle valves and a pressure gauge to control the gas flow and its static pressure. Density and viscosity were measured for CO_2_, N_2_, and air of different pressures in the gas chamber. The vacuum pressure was set using a vacuum pump (Rocker610, Rocker Scientific, New Taipei, Taiwan).

The density and viscosity of deionized water, acetone, and 25% *v*/*v* glycerol were measured using the microscale-thickness cantilever. The cantilever chip with the PZT chip was fixed on a slide glass, and a syringe needle (22 G; inner diameter: 0.4 mm) which contains a liquid inside, was set to slide parallel to the cantilever length direction to adjust the immersion length (Figure 2b). The cantilever was coated with a thin film of aluminum by the manufacturer, and there was no visible capillary flow on the surface when the liquid meniscus was formed on the surface of the cantilever. Although the immersion length was manually controlled and can be changed by evaporation of the liquid, a single measurement was carried out for 2 s and was not affected by the change. After the immersion experiment with a liquid, the cantilever was washed with flowing 70% ethanol and deionized water for 1 min, and then gently dried with a nitrogen gun.

## 4. Results and Discussion

**Figure 3:**
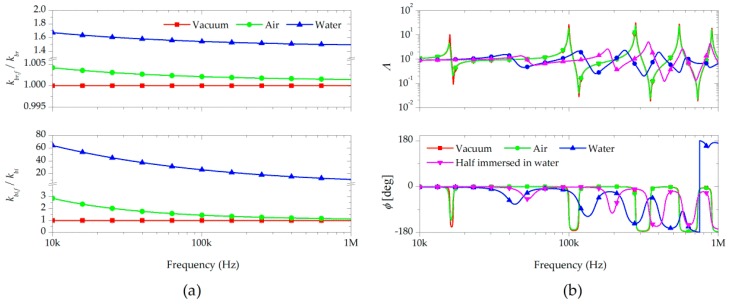
(**a**) Real part (top) and imaginary part (bottom) of the wavenumber ratio of a fluid to vacuum for vibrations of the cantilever in vacuum, air, and water; (**b**) Amplitude (top) and phase (bottom) of the predicted transfer functions of the micro-cantilevers fully immersed in vacuum, air, and water, and half-immersed in water.

For the proposed experimental setup, the effect of the fluid-structure interaction on the wave propagation of the microscale-thickness cantilever was estimated. The fluid loading should have significant impacts on the vibration response for fluid property measurements. If the loading is too large, the vibration occurs as for a completely damped beam without any information about the fluid properties. Figure 3a shows the ratios of the wavenumbers in air and water predicted using Equation (5). The wavenumbers showed greater change in liquid than in gas. As frequency increased, the ratios of the wavenumbers decreased. The real part of the wavenumber affects the wavelength, and the imaginary part influences the vibration amplitude of the cantilever. The liquid densities and viscosities were clearly larger than those of gases, and had more significant influence on the wavenumber.

Vibration responses in the frequency range of the cantilever were determined by the boundary conditions (Equation (6)), and then the transfer function was calculated by Equation (7). The transfer functions of the cantilevers in vacuum and water (completely and partially immersed) are shown in Figure 3b. A significant decrease of the resonance frequencies and the vibration magnitude of the transfer functions occurred in the cantilevers in the water. The change of the transfer function of the cantilever in air was negligible, because the bending stiffness of the cantilever was too large to detect the fine change induced by air. The resonance frequencies and vibration amplitude of the cantilever fully immersed in vacuum and water were different.

**Figure 4:**
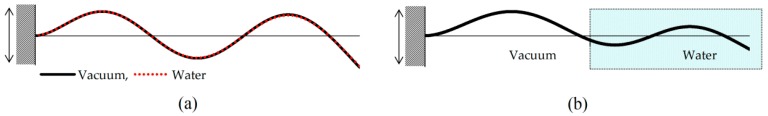
The standing wave pattern at the fourth resonance frequency of the cantilever when (**a**) fully immersed in vacuum and water and (**b**) half-immersed in water.

Figure 4 shows the standing wave pattern at the fourth resonance frequency calculated from Equation (4) when the cantilever was fully and half-immersed in vacuum and water. The standing wave pattern was not affected by the fluid-structure interaction when fully immersed (Figure 4a). On the other hand, the half-immersed cantilever exhibited a different standing wave pattern due to the variation of the wavelength at a single frequency (Figure 4b). This half-immersion was advantageous in reducing the fluid structure interaction, thereby increasing the magnitude of responses required for precise vibration sensing.

**Figure 5:**
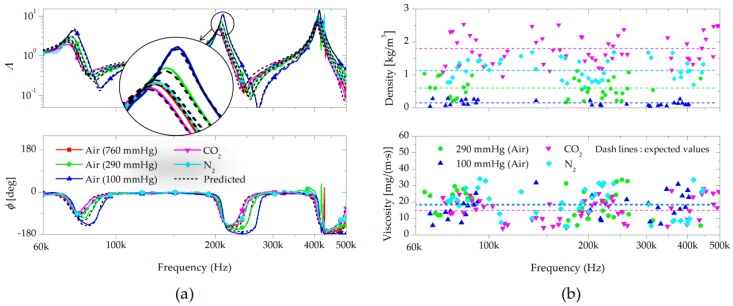
(**a**) Amplitude and phase of the transfer function for the nanoscale-thickness cantilevers under the fluid-structure interaction in air (760 mmHg, 290 mmHg, and 100 mmHg), carbon dioxide (760 mmHg), and nitrogen (760 mmHg), with their predicted values; (**b**) Measured density and viscosity of the gases at different frequencies.

The effects of the fluid-structure interaction on the wave propagation of the cantilever were analyzed by the change in the wavenumber. Figure 5a shows the measured and predicted transfer functions when the cantilever was completely immersed ($l_{g}=0$) in air at different pressures (760 mmHg, 290 mmHg, and 100 mmHg), carbon dioxide at atmospheric pressure, and nitrogen at atmospheric pressure. As shown in Figure 5a, the measured vibration behaviors showed excellent agreement with those predicted by Equation (7). The small change in the transfer function affected by the gas-structure interaction force was measured by utilizing the nanoscale-thickness cantilevers of small bending stiffness. The natural frequencies decreased due to the fluid loading effects, and the vibration damping decreased with increasing frequency.

To measure the dynamic stiffness of the cantilever, air at 760 mmHg was used for a reference fluid, as it has known values of density and viscosity. For the calculation of the density and viscosity, the wavenumbers of the cantilever in the fluids were applied to the proposed process. The densities and viscosities (Figure 5b) were measured at each frequency of the vibration measurements. Although the vibration response showed a significant amount of variations over a wide range of frequencies, the estimated values were almost frequency-invariant in the frequency bands. The obtained fluid properties were averaged out at the mean values, which showed more robustness with more data than the resonance-based methods. The measured densities and viscosities along with typical values are shown in Table 1, where the relative differences between these two varied from 1.6 to 7.3%.

**Figure 6:**
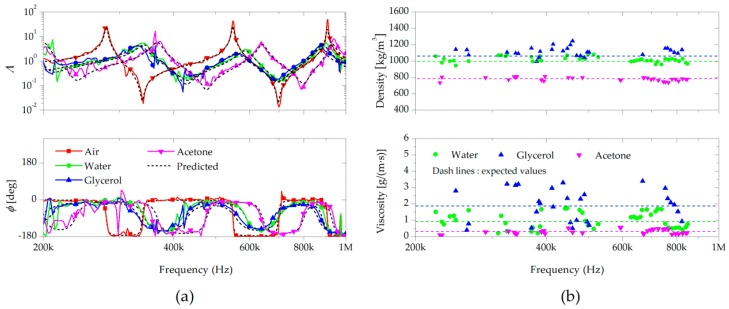
(**a**) Amplitude and phase of the transfer function for the microscale-thickness cantilevers under the fluid-structure interaction in air (760 mmHg), water, 25% (*v*/*v*) glycerol, and acetone, with their predicted values; (**b**) Measured density and viscosity of the liquids at different frequencies.

Figure 6a shows the measured transfer functions when the cantilever was partially immersed in water, 25% (*v*/*v*) glycerol in water, or acetone ($l_{g}=l_{f}=L/2$, where *L* is the length of the cantilever). The fluid-structure interaction reduced the wave speed significantly since the densities of liquids are three orders of magnitude higher than those of gases. The vibration amplitude in liquids was lower than that in vacuum due to the larger dissipation of the wave energy. In fact, the resonant vibration responses were very small when completely immersed in liquids. As the cantilever length in liquid decreased, the natural frequency and overall vibration amplitude increased. Therefore, the cantilevers were controlled to be partially immersed so that the vibration resonance was sufficient for measuring cantilever oscillation and its interaction with the surrounding fluids. The influence of fluid-structure interaction was significant at low frequencies, and decreased with increasing frequency as predicted by Equation (5). The wavenumber variation also decreased for the cantilever in vacuum with increasing frequency.

Figure 6b shows the calculated density and viscosity after solving for the flexural wave interaction with the partial immersions. When the cantilevers were immersed in the liquids, the variation of the transfer functions due to the fluid loading was significantly larger than those in the gas. With the larger influence of the fluid loading, the measured liquid densities showed smaller variations than those of the gases, which may be due to the reduced relative influence from sensitivity of the sensor and noise due to external flow. The mean values of the measured properties and its comparison to the properties obtained from the literature are shown in Table 2. With the partially immersed cantilevers, the discrepancies between the measurements and typical values were less than 4.8% for the densities and 3.1% for the viscosities. The accuracies for the viscosities was better than previous studies that had used a change in resonant frequency and peak width for the measurements [1,3,4,5,7,8,9,10]. Moreover, the measurement was more accurate in liquids than in gases, which is interesting, because in most resonant sensors, it is easier to make measurements in gases because of the larger quality factors in gases. This increased accuracy was due to the larger fluid loading effects on the estimated wavenumber. The accuracy also depended strongly on the frequency ranges and cantilever properties. Therefore, if the range of fluid properties is known beforehand, the cantilever specifications can be optimized to maximize the fluid-structure interaction in the frequency range of the data acquisition, thereby increasing the accuracy.

## 5. Conclusions

We have presented an alternative method to measure the density and viscosity of liquids and gases using a wave propagation analysis on microscale- and nanoscale-thickness cantilevers. The microscale-thickness cantilevers were partially immersed in liquids to achieve sufficient vibration resonance. With this configuration, the wave propagations were measured to determine the interaction between the cantilever and the fluid, providing the exact vibration solution of the cantilever, and hence the density and viscosity of the fluids. In the proposed method, the distributed fluid loading was analyzed in a wide frequency range of measurements, whereas previously an oscillating sphere of an effective radius for a specific mode was assumed to analyze the influence of the fluid loading. The exact solution of the cantilever vibration without prior information on the vibration mode was advantageous for securing the flexibility of the experimental setup related to the immersed length in the testing liquid. This method also allowed for the precise and robust determination of the surrounding fluid properties with minimal influence from external noise because the transfer function was obtained from the relative displacement between two locations on the cantilever. The wave approach for the vibration analysis allows for the verification of the experimental setup since defective parts or improper boundary conditions induce completely different vibration behaviors [21]. The natural frequencies were not required for these measurements. In the present study, the measurement was performed using a laser vibrometer. The vibration generation was performed using piezo excitation. When the vibration sensing is performed using piezo or capacitive effects, integrated sensor fabrication is possible.

## Figures and Tables

**Table 1:** Mean values and standard deviations of the measured densities and viscosities of the gases at 27 °C and their comparison to the typical values from the literature [17,18].

Gas	Density (kg/m^3^)		Viscosity (×10^−5^ kg/(m·s))	
	Expected	Measured	Expected	Measured
Air, 760 mmHg	1.18	-	1.86	-
Air, 290 mmHg	0.60	0.59 ± 0.06	1.86	1.82 ± 0.17
Air, 100 mmHg	0.15	0.14 ± 0.02	1.86	1.83 ± 0.18
CO_2_, 760 mmHg	1.80	1.75 ± 0.08	1.50	1.61 ± 0.12
N_2_, 760 mmHg	1.14	1.09 ± 0.07	1.80	1.93 ± 0.20

**Table 2:** Mean values and standard deviations of the measured densities and viscosities of the liquids at 23 °C and their comparison to the expected values from the literature [19,20].

Liquid	Density (kg/m^3^)		Viscosity (×10^−3^ kg/(m·s))	
	Expected	Measured	Expected	Measured
Water	997.3	1016.8 ± 6.1	0.935	0.943 ± 0.082
25% Glycerol	1063.5	1114.3 ± 12.6	1.887	1.946 ± 0.197
Acetone	787.7	773.9 ± 4.5	0.311	0.313 ± 0.032

## References

[1] Badarlis A., Pfau A., Kalfas A. (2015). Measurement and evaluation of the gas density and viscosity of pure gases and mixtures using a micro-cantilever beam. Sensors.

[2] Cakmak O., Ermek E., Kilinc N., Bulut S., Baris I., Kavakli I., Yaralioglu G., Urey H. (2015). A cartridge based sensor array platform for multiple coagulation measurements from plasma. Lab Chip.

[3] Cakmak O., Ermek E., Kilinc N., Yaralioglu G., Urey H. (2015). Precision density and viscosity measurement using two cantilevers with different widths. Sens. Actuators A Phys..

[4] Wilson T.L., Campbell G.A., Mutharasan R. (2007). Viscosity and density values from excitation level response of piezoelectric-excited cantilever sensors. Sens. Actuators A Phys..

[5] McLoughlin N., Lee S.L., Hähner G. (2007). Temperature dependence of viscosity and density of viscous liquids determined from thermal noise spectra of uncalibrated atomic force microscope cantilevers. Lab Chip.

[6] Quist A., Chand A., Ramachandran S., Cohen D., Lal R. (2006). Piezoresistive cantilever based nanoflow and viscosity sensor for microchannels. Lab Chip.

[7] Papi M., Arcovito G., De Spirito M., Vassalli M., Tiribilli B. (2006). Fluid viscosity determination by means of uncalibrated atomic force microscopy cantilevers. Appl. Phys. Lett..

[8] Agoston A., Keplinger F., Jakoby B. (2005). Evaluation of a vibrating micromachined cantilever sensor for measuring the viscosity of complex organic liquids. Sens. Actuators A Phys..

[9] Shih W.Y., Li X., Gu H., Shih W.-H., Aksay I.A. (2001). Simultaneous liquid viscosity and density determination with piezoelectric unimorph cantilevers. J. Appl. Phys..

[10] Bergaud C., Nicu L. (2000). Viscosity measurements based on experimental investigations of composite cantilever beam eigenfrequencies in viscous media. Rev. Sci. Instrum..

[11] Sader J.E. (1998). Frequency response of cantilever beams immersed in viscous fluids with applications to the atomic force microscope. J. Appl. Phys..

[12] Hong S., Kim D., Park J., Jang J. (2015). Simultaneous position and mass determination of a nanoscale-thickness cantilever sensor in viscous fluids. Appl. Phys. Lett..

[13] Tuck E.O. (1969). Calculation of unsteady flows due to small motions of cylinders in a viscous fluid. J. Eng. Math..

[14] Landau L. (1987). Em Lifshitz Fluid Dynamics.

[15] Fahy F.J. (2012). Sound and Structural Vibration: Radiation, Transmission and Response.

[16] Park J. (2005). Transfer function methods to measure dynamic mechanical properties of complex structures. J. Sound Vib..

[17] Lienhard J.H. (2013). A Heat Transfer Textbook.

[18] Hinds W.C. (2012). Aerosol Technology: Properties, Behavior, and Measurement of Airborne Particles.

[19] Cheng N.-S. (2008). Formula for the viscosity of a glycerol—Water mixture. Ind. Eng. Chem. Res..

[20] Peng I.H., Tu C.H. (2002). Densities and viscosities of acetone, diisopropyl ether, ethanol, and methyl ethyl ketone with a five-component hydrocarbon mixture from 288.15 k to 308.15 k. J. Chem. Eng. Data.

[21] Lee S., Jeong S., Park J. (2014). Damage identification using flexural vibration actuated and sensed by piezoelectric transducers. Proc. Inst. Mech. Eng. Part C J. Mech. Eng. Sci..

